# Changes of Soybean Protein during Tofu Processing

**DOI:** 10.3390/foods10071594

**Published:** 2021-07-09

**Authors:** Xiangfei Guan, Xuequn Zhong, Yuhao Lu, Xin Du, Rui Jia, Hansheng Li, Minlian Zhang

**Affiliations:** 1 Department of Chemical Engineering, Institute of Biochemical Engineering, Tsinghua University, Beijing 100084, China; gxf0413@163.com (X.G.); xq.behappy@gmail.com (X.Z.); luyh17@tsinghua.org.cn (Y.L.); du-x18@mails.tinghua.edu.cn (X.D.); jiar19@mails.tsinghua.edu.cn (R.J.); 2 School of Chemistry and Chemical Engineering, Beijing Institute of Technology, Beijing 102488, China; hanshengli@bit.edu.cn

**Keywords:** tofu, protein, structure, mechanism

## Abstract

Tofu has a long history of use and is rich in high-quality plant protein; however, its production process is relatively complicated. The tofu production process includes soybean pretreatment, soaking, grinding, boiling, pulping, pressing, and packing. Every step in this process has an impact on the soy protein and, ultimately, affects the quality of the tofu. Furthermore, soy protein gel is the basis for the formation of soy curd. This review summarizes the series of changes in the composition and structure of soy protein that occur during the processing of tofu (specifically, during the pressing, preservation, and packaging steps) and the effects of soybean varieties, storage conditions, soybean milk pretreatment, and coagulant types on the structure of soybean protein and the quality of tofu. Finally, we highlight the advantages and limitations of current research and provide directions for future research in tofu production. This review is aimed at providing a reference for research into and improvement of the production of tofu.

## 1. Introduction

Tofu originated in the time of the Western Han dynasty and has been consumed for more than two thousand years. Tofu is rich in soy protein and has high nutritional value [1]. Processing of soy products can remove most of the anti-nutritional factors in soy and significantly improve the digestibility of soy protein. Studies have shown that the digestibility of whole ripe soybeans is only 65.3%; after processing it into soy milk and tofu, the digestibility becomes 85% and 92–98%, respectively [2]. Furthermore, the FDA authorized a “Soy Protein Health Claim” on 26 October 1999 stating that 25 g of soy protein a day may reduce the risk of heart disease. In addition to protein, tofu contains lipids, carbohydrates, crude fiber, isoflavones, minerals, and saponins, which can lower cholesterol, alleviate the symptoms of cardiovascular and kidney diseases, and reduce the incidence of cancer and tumors [3].

Several types of tofu are available in the market to meet the different needs of consumers, each produced via a different complex process. Here, we review the changes that occur in soybean protein in each step of tofu production (specifically, during soaking and refining). The types, composition, and structure of soy protein and the effects of soy varieties and storage conditions on soy protein are summarized. In addition, the effects of pretreatment steps and coagulants on soy protein structure and tofu quality are introduced. We also summarize the advantages and disadvantages of current research and provide directions for future research into soy protein structural changes during tofu processing. Overall, this review is expected to serve as a foundation for research into the curdling mechanism of tofu and as a theoretical guide for the actual production process.

## 2. Soy Protein

**Figure 1:**
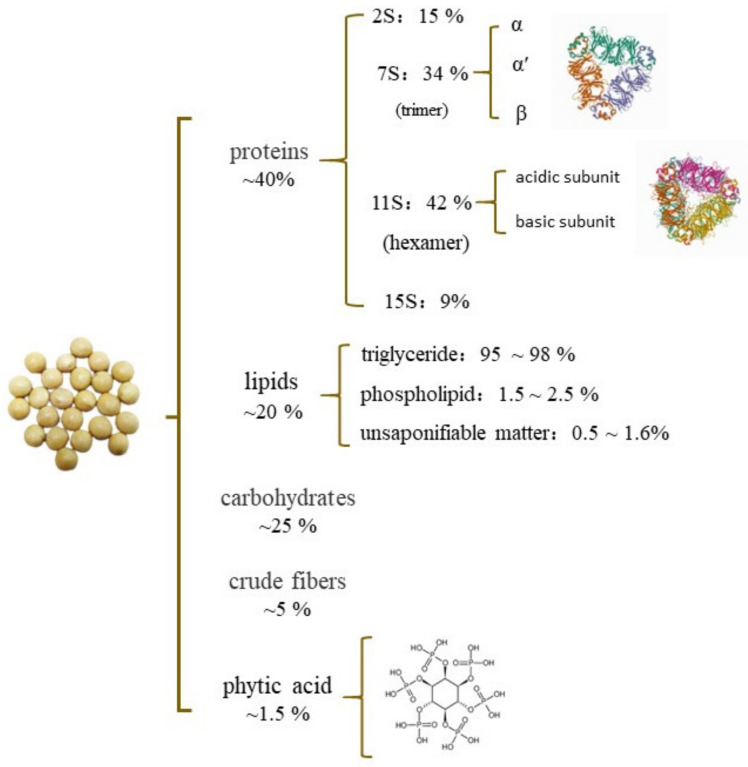
Nutritional composition of soybean [5,6]; Protein Data Bank.

Soy is composed of approximately 40% proteins, 20% lipids, 25% carbohydrates, and 5% crude fibers [2,4]. They are also rich sources of isoflavones, minerals, and other components. The nutrient contents of soybean are shown in Figure 1.

Soy protein can be classified according to its solubility, physiological function, and centrifugal sedimentation speed. Based on solubility, soy protein is divided into globulin and albumin. Based on physiological functions, it is divided into storage protein and biologically active protein. Based on centrifugal sedimentation speed, it is divided into 2S, 7S, 11S, and 15S components (S refers to the sedimentation coefficient, where 1S = 10^−13^ s = 1 Svedberg), and each component is composed of protein molecules with similar molecular weights [4]. The graded composition of soy protein is shown in Table 1.

The 7S conglycinin and 11S glycinin are the key components of tofu curd, accounting for more than 70% of the total soy protein content [11]. 7S conglycinin is a trimer composed of an α subunit, α′ subunit, and β subunit and accounts for approximately 30% of the soy protein content [12]. 11S glycinin is a hexamer composed of acidic polypeptides (A) and basic polypeptides (B) linked by disulfide bonds and accounts for approximately 40% of the soy protein content. The composition and functions of 7S conglycinin and 11S glycinin are shown in Table 2. Ren et al. [13] analyzed the interaction between protein subunits in soy milk and proposed a model for protein subunit structure. The β subunit and the B subunit form the hydrophobic core of the granule protein through electrostatic interaction, in which the B subunit is covalently connected by disulfide bonds. Other hydrophilic subunits, such as α, α’, and A subunits, are distributed around this hydrophobic core through hydrophobic interactions and hydrogen bonds.

The functional properties of soy protein mainly include gelling, emulsification, and foaming; of these, gelling is the basis for the formation of tofu. During tofu production, soy protein undergoes dissociation or association reactions during the acid-base treatment and heat treatment, which changes the ionic strength of the solution. Through these association–dissociation reactions, 11S glycinin polymerizes to form dimers, oligomers, or multimers or dissociates to form 7S and 3S components [14].

## 3. Changes in Soy Protein during Tofu Processing

**Figure 2:**
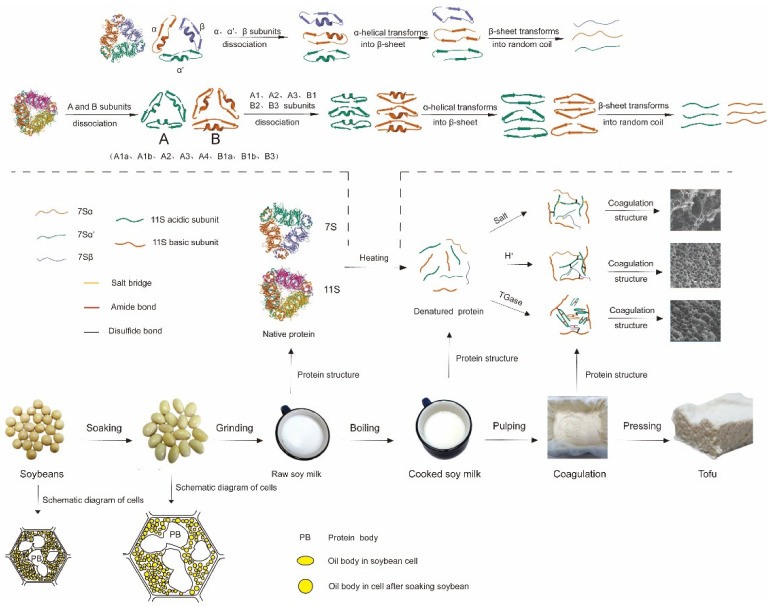
Changes of soybean protein during tofu processing [5,6]; Protein Data Bank.

To obtain raw soy milk for tofu production, soybeans are soaked, pulped, and filtered. For tofu production using the raw soy milk, the milk is first heated and then a coagulant is added to form tofu curd. The curd is then pressed to obtain a tofu product. As shown in Figure 2, the structure and content of soy protein undergo numerous changes throughout the tofu production process. The quality of the final tofu product is affected by soybean varieties, storage conditions, soaking, grinding, soymilk pretreatment, types of coagulants, operating conditions, pressing, and packaging.

### 3.1. Influence of Soybean Varieties and Growing Conditions

The protein, the 11S/7S ratio, and the methionine and cysteine content in soybeans have a significant impact on the hardness and the water-holding capacity of tofu [17]. Therefore, these indices are important indicators for screening soybeans [18].

Different soybean varieties differ in their soy protein subunit compositions, leading to changes in their denaturation temperature and gel network structure [19,20,21]. Stanojevic [22] and Cai et al. [23] assessed the effects of soybean varieties on the quality of tofu. They found that soybeans with a low 11S/7S ratio formed a uniform spherical aggregated gel, whereas the gel formed by beans with a higher 11S/7S ratio had higher macroscopic phase separation, a coarser network structure, and larger pores [24].

The content of the 11SA4 subunit in soybean can affect the overall protein content and seed size. Soybeans with lower 11SA4 content have higher protein content and smaller seeds, and the gel structure of the obtained tofu is denser. Therefore, the protein content in soybeans can be increased by removing the 11SA4 subunit through genetic breeding, thereby improving the gel properties, hardness, and water-holding capacity of the tofu produced [18].

The growth environment and growing period of soybean also affect the 7S and 11S content and the composition of their subunits. The total protein content in soybeans is negatively correlated with latitude (12–32 N°) and rainfall in the growing season (61–956 mm) and positively correlated with the daily average temperature in the growing period (19.0–26.7 °C) [25]. Yang et al. [26] showed that soybeans from different growth environments presented obvious differences in protein subunits, which in turn influenced the yield, color, hardness and water loss of tofu. Poysa et al. [27] observed that the effects of soybean genetic profile and growth year significantly affected soymilk and tofu yield, solids levels, and pH and effected tofu color, hardness, and firmness more than the growth environment. Moreover, the effects of the interaction of genotype with location and year were minor relative to the effects of genotype and year individually.

### 3.2. Influence of Storage Conditions

Generally, the newly harvested soybeans are unripe and have lower oil and protein content than fully mature seeds, making processing difficult. After storage, soybeans mature, resulting in improved tofu yield, color, hardness, and water loss. However, extended storage can also lead to a decline in tofu quality.

The main factors affecting soybean storage include the relative humidity of the storage environment, seed moisture content, storage temperature, and duration. Kong et al. [28] found that long-term storage leads to a decrease in the water-holding capacity of soy protein, thus increasing the yield and protein content of tofu. Saio et al. [29] showed that the relative humidity of the soybean protein storage environment has a greater impact on soybean protein components than the storage temperature.

In summary, different soybean varieties have different genes, protein composition, and 11S/7S protein ratios. The growth environment affects the gene expression of soybeans, which influences the composition and structure of their protein. Storage conditions, on the other hand, cause physical, chemical, and biological changes in soy protein. Therefore, both storage conditions and storage time influence the protein content of tofu.

### 3.3. Influence of Soaking and Refining

Soaking and refining are important steps in tofu processing. Soaking changes the structural characteristics and crushing performance of soybeans, accelerating the extraction of soybean protein and thereby increasing the protein content of tofu [30]. Conversely, the limited swelling of soy protein doubles the absorption of water and increases soybean volume. Refining can dissolve the protein in soybeans and disperse them evenly in water. In the refining process, the extraction rate of soy protein is approximately 85% [2].

The soybean soaking process is affected by several factors, such as soybean particle size and variety, soaking water quality, water temperature, pressure, and soaking method and time. Therefore, choosing the right soaking conditions is crucial in the processing of tofu. High-quality water and suitable temperature and soaking time lead to a higher protein extraction rate and content in soymilk, increasing the gel strength and water-holding capacity of tofu [31,32,33]. Yang et al. [30] and Pan et al. [34] showed that the optimal soaking time decreases as the soaking temperature increases. However, as the temperature rises from 30 °C to 40 °C, protein and carbohydrates leak significantly and the solid content of the soaked soybean seeds decreases. Guo et al. [35] showed that the order of factors affecting the yield of soy protein during the soaking process was soaking time > soaking temperature > pH of the soaking solution. Furthermore, the optimal conditions of soaking may also be affected by soybean varieties.

In conclusion, soaking and refining dissolve the protein, oil, and other substances in soybeans from a solid to liquid phase. The treatment conditions of soaking and refining, such as soaking water quality, soaking water temperature and time, refining temperature, and the material-to-water ratio of refining, affect the dissolution of soy protein, oil, and other components and change the content of each component in soymilk, affecting the quality of tofu.

### 3.4. Pretreatments

#### 3.4.1. Soybean Pretreatment

Okara is rich in dietary fiber, protein, fat, and isoflavones. However, the tofu production process usually removes the okara in soymilk, causing nutrient loss. Therefore, researchers have developed a preparation method that produces whole soybean curd while retaining the okara. However, the dietary fiber, gel, and other impurities contained in soybeans have an adverse effect on the texture and flavor of tofu.

Studies have shown that the protein and oil content of dehulled soybeans is higher than whole soybeans [36]. The use of dehulled soybeans to produce tofu not only improves product quality but also facilitates the extraction of soy protein. Moreover, the network structure of whole bean curd is mostly irregular, discontinuous, large, and uneven. This is because some insoluble dietary fiber particles are embedded in the network structure, which destroys the continuity of the soy protein gel network [37].

Tofu prepared from frozen soybeans showed a more orderly and denser network structure compared to unfrozen soybeans, inducing an increase in some textural parameters, such as hardness, springiness, gumminess, chewiness, and syneresis. Freezing also enhanced tofu quality with a lower yield, lower fat, and higher protein content [38].

#### 3.4.2. Soymilk Pretreatment

Denaturation of soy protein is a prerequisite for curd formation of tofu and is generally applied in the form of heat in tofu processing technology [39]. Studies have found that the hydrophobicity, emulsification, and gel strength of soy protein can be enhanced by ultrasonic treatment of soy milk.

(1) Heat Treatment

Raw soymilk is relatively stable because the natural soybean protein molecule has a hydrophobic group inside and a hydrophilic group on the surface of the molecule. As the raw soymilk is heated, the energy in the system increases, the thermal motion of protein molecules intensifies, and the vibration frequency of certain groups in the molecule increases. This change leads to the breaking of the secondary bonds that hold the protein molecular structure, while the spatial structure also changes [40]. During the thermal denaturation of soy protein, hydrophobic groups such as sulfhydryl groups, disulfide bonds, and hydrophobic amino acid side chains are exposed, and the hydrophobicity of the surface of soy protein increases, which intensifies the protein molecule aggregation.

Soybean protein aggregates during soybean milk heat treatment. At 80 °C, the solubility of 11S globulin decreases, and the α-helical structure of the protein gradually transforms into β-sheet and random coil structures. After heat treatment at 90 °C and 100 °C, the solubility of protein increases slightly, and the α-helix and β-sheet structures change to β-turn and random coil structures, which plays an important role in the formation of aggregates [41,42]. In the formation of thermal aggregates and network structures, β-sheets have a greater effect than α-helixes [43]. The decrease in β-sheet content exposes the hydrophobic area of the protein [44]. Guo et al. [40] showed that 7S produced soluble limited aggregations, while 11S formed insoluble aggregations. After heating, 7S terminated the assembly of 11S and restored the solubility of 11S. The three-dimensional network structure of agglomerates prepared by heated soybean protein shows low sedimentation and a high curdling rate, suitable water-holding capacity, low hardness, and high elasticity.

Currently, tofu is prepared with soymilk at approximately 100 °C. Nevertheless, as the heat denaturation temperature of 11S protein (85~95 °C) is estimated to be 20 °C higher than the heat denaturation temperature of 7S protein [45], such a heating method denatures both proteins almost simultaneously. Studies have shown that the two-step heating method (i.e., first denaturing 7S protein, then 11S protein) is conducive to the effective denaturation of soy protein and the formation of curd, maximizing the use of the two-storage protein gel characteristics to obtain the best quality curd [46].

During the heating process, in addition to the structural changes of the protein itself, the oil in the soymilk also has a certain impact. Peng et al. [47] described this process in detail. Between 65–75 °C, oil molecules, 7S, and 11S are released into soluble components from the storage protein–oil body complex. At 75–95 °C, the oil enters the floating components on the surface of the soymilk. The β and B subunits then aggregate to form protein particles, while the α, α’, and A subunits remain in the soluble fraction.

Heating induces protein aggregation and protein–polysaccharide interaction, leading to the modification of protein particle size distribution, viscosity, surface hydrophobicity, and solubility [48] and altering the structure of soybean protein. Moreover, soy protein has hydrophobic interactions with flavor substances, such as aldehydes and ketones. In addition to the aggregation of subunits, the conformation of the polypeptide chain also varies during thermal denaturation. The exposed hydrophobic areas on the surface of the heat-denatured protein particles provide active binding sites for flavor molecules and affect the sensory quality of soymilk [49].

(2) Ultrasound Pretreatment

Ultrasound is a phenomenon of cavitation that exceeds the threshold of human hearing. The cavitation effect of ultrasound can change the structure of protein molecules. After ultrasonic treatment, the polypeptide chain inside the protein molecule is partially expanded, the protein structure becomes more stretched, the hydrophobic group is exposed, the surface activity enhances, and the emulsification increases [50].

Liu et al. [51] found that the 11S globulin aggregates were broken into small uniform particles after ultrasonic treatment, which narrowed the distribution and increased the surface charge density. Proteins can be completely distributed in the oil–water interface by appropriate ultrasound and heat treatment, thus reducing surface tension. As the sonication time extends, the emulsification of soy protein tends to be stable after its peak [52]. In this regard, Chen et al. [53] and Karki et al. [54] suggested that with a sonication time extension the protein structure becomes loose, the polar part shifts to water, and the non-polar part shifts to lipids. The emulsion becomes evenly dispersed and the emulsification performance is improved. However, if it is processed for a long time, the insoluble protein content increases and the emulsification decreases.

Different ultrasound powers have different effects on proteins. Liu et al. [55] found that low-power ultrasound treatment weakens the ability of soy protein gel formation, and high-power ultrasound delays the formation of the gel. However, under the treatment of medium-power ultrasound (200–600 W), the hydrophobic interaction and hydrogen bonding positions during the formation of the thermal gel increase, forming a stronger gel with a denser three-dimensional network structure. This improves the gel properties of soy protein. Li et al. [56] found that with the increase of ultrasound power, the particle size of soy protein gradually increased, reaching the highest value at 300 W. However, if the ultrasonic power continued to be enhanced, the particle size of soybean protein decreased, hydrophobic groups were exposed, and the emulsification and stability of soybean protein were improved.

Hu et al. [57] found that with ultrasonic treatment under 400 W, the calcium ion-induced soy protein formed a compact and uniform three-dimensional gel structure, which improved its water-holding capacity and gel strength. Zhang et al. [58] showed that high-intensity ultrasound can open the spatial structure of soy protein isolate, exposing the site of action of transglutaminase, thus enhancing the strength of the gel formation induced. Therefore, the modification of soy protein by ultrasound can be applied to tofu processing.

### 3.5. Coagulants

A key step in tofu production is the addition of a coagulant to make the soybean protein form a gel network structure that is macroscopically reflected in the formation of tofu coagulation. This process is mainly influenced by the type of coagulant and processing conditions. During the coagulation process, the protein–protein and protein–water interactions cause soy protein to aggregate and form a honeycomb-like gel [59]. Currently, typical coagulants include salt, acid, and enzyme and composite coagulants. They have different coagulation mechanisms and interfere with the quality of tofu.

#### 3.5.1. Salt Coagulants

Salt coagulants are the most traditional and widely used in tofu production; mainly include magnesium chloride, calcium chloride, magnesium sulfate, calcium sulfate, calcium acetate, and so on. Regarding the curdling mechanism of salt-based coagulants to make tofu, researchers believe that the gelatinization process of tofu can be divided into two steps [60]: (1) the heat denaturation process of protein and (2) a hydrophobic condensation process promoted by metal ions.

There are three main theories for the mechanism of salt coagulants: (1) ion bridge theory [61], (2) salting-out theory [62], and (3) isoelectric point theory [63]. In recent years, researchers have also proposed a new explanation based on the ion bridge theory, which states that the formation of protein particles varies in the presence of specific metal ions [64]. These four explanations, however, have their limitations.

In order to explore the solidification mechanism of salt coagulants, researchers have studied the contributions of various interaction forces in the solidification process. Lee et al. [61] used optical and scanning electron microscopy to observe the microstructure of soy protein aggregates during heat treatment and spotting. They found that the isoelectric point precipitation and calcium ion aggregation did not change the globular structure of soy protein, but heating could change the protein structure. Zhou et al. [65] studied the changes in the texture characteristics of the gel by adding different types of additives, including NaCl, thiothreitol, sodium dodecyl sulfate, and urea, during the preparation of the marinated tofu gel. Their results showed that electrostatic interactions, disulfide bonds, hydrophobic interactions, and hydrogen bonds have important effects on the formation of tofu gel. Liu et al. [66] analyzed freeze-dried tofu samples using chemical methods and studied the influence of intermolecular forces in the curding process of different coagulants on this basis. They highlighted that hydrophobic interaction and disulfide bonds play a dominant role in the formation of soybean gel. Yang et al. [67] studied the changes in the secondary structure and moisture state of the protein during the formation of tofu gel and concluded that electrostatic interaction and hydrophobic interaction mainly affect the aggregation of protein molecular chains; moreover, hydrogen bonds and disulfide bonds mainly affect the connection of molecular chains. Jin et al. [68] showed that as the solidification temperature increases, the proportions of ionic and hydrogen bonds decrease significantly, while the proportions of hydrophobic interactions and disulfide bonds increase. In summary, hydrogen and disulfide bonds and electrostatic and hydrophobic interactions play a certain role in the curdling process of tofu, but there are still some controversies about their specific mode of action.

The type and concentration of salt coagulants play a decisive role in the properties of tofu curd. Lu et al. [63] found that a variety of calcium salts (calcium chloride, calcium lactate, calcium acetate, calcium gluconate, etc.) can induce gelation of soy protein; thus, it precipitated when the pH of soymilk was 6. Liu et al. [69] found that as the concentration of coagulant increased, the gel strength of tofu increased, while the water retention decreased.

The type of ions affects the coagulation characteristics of tofu, and the influence of anions is greater than that of cations. Other than soy protein, lipids are also the main components of soymilk. With the addition of the coagulant, the lipid particles incorporate into the protein gel network and disperse in the gel network. Based on previous research, Peng et al. [47] proposed a specific model that combines soy protein, lipids, and small molecules.

#### 3.5.2. Acid Coagulant

Acid coagulants are another important type of tofu coagulants. Acid coagulants include glucolactone (GDL), physalis, lactic acid, acetic acid, succinic acid, citric acid, malic acid, and tartaric acid; of these, GDL is the most widely used. Acid coagulants can provide hydrogen ions that reduce the pH value of soymilk to the isoelectric point of soy protein, thus promoting the isoelectric precipitation of protein.

At a certain temperature, GDL slowly hydrolyzes the gluconic acid and releases protons, which is a suitably gradual process for forming a continuous soy protein network structure through hydrophobic and electrostatic interactions [70]. Excessive gel rate can result in gel with an uneven structure and low strength [71]. Kaoru et al. [60] showed that the gelation process of GDL is similar to that of salt-based coagulants, and its gelation curve conforms to first-order reaction kinetics. However, the coagulation rate induced by GDL is lower than that of salt-based coagulants [60]. Due to the difference in isoelectric point, there are more protons recombined in 7S than 11S. The addition of GDL promotes aggregation through hydrophobic interactions, thereby inducing gelation, whereas the interaction between charges may be secondary.

During the tofu production process, yellow water containing certain nutrients is produced. Under suitable conditions, the yellow water can be fermented to obtain physalis. Using physalis as a coagulant can save costs and reduce pollution. Liu et al. [72] showed that the physalis coagulant produces a large amount of hydrogen ions. The ions reduce the negative charge of protein molecules, increase the content of free sulfhydryl groups, and gradually reduce the surface hydrophobicity, thus inducing the formation of protein aggregates.

#### 3.5.3. Enzyme Coagulant

Coagulant enzymes, widely present in animal and plant tissues and microorganisms, have great development potential as bean curd coagulants. At present, the most studied enzyme coagulants include transglutaminase (TGase), pepsin, acalase, papain, and bromelain.

From the 1980s to the beginning of this century, researchers tried to use natural proteases derived from plants and animals as a tofu coagulant. Fuke et al. [73] confirmed the role of bromelain in the aggregation and gelation of heated soymilk by measuring sulfhydryl content and hydrophobicity. Luan et al. [74,75] studied 13 different proteases and found that alcalase, papain, and bromelain have a strong soymilk solidifying ability.

TGase is a type of enzyme that catalyzes the acyl transfer reaction between the γ-hydroxylamine group (acyl donor) of peptide glutamine residues and a variety of primary amines (acyl acceptor). This process is mainly realized in three ways: introduction of amines, intermolecular and intramolecular cross-linking, and deamination. Yang et al. [76] believed that the effect of TGase in improving the strength of tofu is mainly related to 7S and 11S protein. In this process, the α’ and α subunits in 7S and the A3 peptide chain in 11S have the greatest influence on the action of TGase, followed by the β subunit in 7S and the A peptide chain in 11S. They analyzed the amino acid content of these subunits and peptide chains and concluded that the activity of TGase is closely related to the lysine in soy protein.

#### 3.5.4. New Coagulants

(1) Emulsion Coagulant

Magnesium chloride has high solubility and can be used in a rapid and intense tofu gelation process. The coagulation rate of tofu curd influences the characteristics of the curd. Overly quick coagulation results in coagulation with poor water retention and coarse particles. As a slow-release platform, emulsion coagulant can achieve controlled release of coagulant, solving the above problems [77,78]. The main components of emulsion coagulant include a brine-based water phase, a natural fat-based oil phase, emulsifier, and protein. The current emulsion coagulants can be divided into two types: water-in-oil (W/O) and water-in-oil-in-water (W/O/W).

Li et al. [79,80] studied the preparation methods of W/O and W/O/W emulsion coagulants and their influence on the characteristics of tofu curd and found that the emulsion coagulant can improve the spatial structure of tofu gel. Compared with the traditional marinated tofu, the tofu gel network prepared by the emulsion coagulant is finer, and its water content and water-holding capacity are improved. Zhu et al. [81] used a differential calorimetry scanner and low-field nuclear magnetic resonance to study the change of water in the W/O tofu gel process and found that tofu prepared using this coagulant had higher free water content than did traditional marinated tofu.

(2) Complex Coagulants

Common coagulants such as gypsum, brine, and GDL present disadvantages compared to other coagulants. The coagulation speed using a small amount of gypsum is slow, whereas tofu products made with a large amount of gypsum contain residues and a bitter taste. Tofu made with bittern has poor water-holding capacity, resulting in short shelf life. Lactone tofu is soft, and not suitable for frying [82,83,84]. Obtaining a complex coagulant that can overcome the shortcomings of a single coagulant and ensure the quality and taste of tofu has become a major research focus in the field.

Different coagulants have different abilities to induce the coagulation of soy protein, and the ratio of complex coagulants also influences the aggregation state and gel properties of the protein. Wang et al. [85] used magnesium sulfate and calcium sulfate as a complex coagulant to induce gelation of the soybean protein emulsion. The results showed that different concentrations of magnesium ions changed the hardness and strength of the gel due to different aggregation forces. At low concentration, magnesium ions were conducive to the formation of dense protein aggregates and promoted the uniformity and deformation resistance of the gel; when the concentration of magnesium ions increased, the synergistic effect of calcium and magnesium ions promoted the coarsening of the protein gel structure, and the emulsification performance was significantly improved.

Ramy et al. [86] added nano fish bones to the citric acid-induced soymilk curdling system, which significantly enhanced the compactness and uniformity of the three-dimensional gel network. The combination of citric acid and salt coagulants increases the hardness of tofu, probably due to the increase of ionic bonds caused by the addition of salt ions. Salt can change the structures of water and polar groups and provide electric charge, affecting the electrostatic and hydrophobic interactions [87]. The complex coagulant formed by TGase and lactic acid bacteria can induce the formation of a denser gel network [88]. Shi et al. [89] used a composite coagulant of TGase and GDL to prepare tofu, resulting in enhanced water content, water-holding capacity, and microstructure density under certain operating conditions.

#### 3.5.5. Additives

(1) Carbohydrates

Adding carbohydrates and other additives to the coagulant can significantly improve the performance of tofu curd. The interaction between polysaccharides and protein polymers has been shown to effectively improve the properties of the curd [90]. Common carbohydrate additives include chitosan, guar gum, carrageenan, acacia gum, and konjac gum.

Li et al. [90] compared the tofu made with a compound coagulant (magnesium chloride and guar gum) with traditional tofu (gypsum and marinated tofu) and found that the addition of guar gum affected the gel structure and texture characteristics of tofu. Researchers speculated that the higher viscosity of guar gum increases soymilk viscosity, resulting in a slower coagulation rate. In addition, they compared the performance of carrageenan, guar gum, and acacia gum mixed with magnesium chloride to make tofu [91]. The texture data show that the addition of guar gum significantly reduced the hardness and protein content of tofu, and carrageenan increased the hardness of the curd, while the protein content remained unchanged.

Cao [92] showed that salt coagulants and polysaccharides have a synergistic promotive effect on soy protein curd formation, which significantly improves the texture properties of tofu. Zhao et al. [93] added konjac gum, gellan gum, and Kotlan gum to the calcium sulfate-induced soy protein isolate gel system and found that the addition of polysaccharides enhanced the gel structure, accelerated gelation, improved the microstructure of the gel, and lowered the starting temperature of gelation. Chitosan as a coagulant lowers tofu’s ash content and improves its protein content [94], and in the production process of pressurized lactone tofu, improves its water holding capacity [95]. Jun et al. [96] used acetic acid-treated crab shell extract as a coagulant, and the texture of tofu produced was comparable to that of commercially available tofu.

(2) Phytic Acid

Phytic acid or phytate added to soymilk has a significant effect on the texture characteristics of tofu curd. Schaefer et al. [97] studied the relationship between the content of various components in soybeans and the properties of tofu. They concluded that phytic acid can preferentially combine with calcium coagulants, thereby changing the yield, composition, texture, and microstructure of tofu curd. Saio et al. [98] found that when the amount of calcium salt added was in a certain range, as the phytic acid content in soymilk increased, the effect of calcium ions on protein coagulation decreased and tofu gel formation became increasingly difficult. Therefore, tofu made from soymilk with high phytic acid content has a high yield but low gel hardness. Ishiguro et al. [99] performed curdling experiments and measured the phytic acid content of 27 types of soybeans. The results showed that tofu with a higher phytic acid content and prepared at a general coagulant range had a softer texture. In conclusion, the higher the phytic acid content during the curding process, the lower the hardness of the tofu and the higher the viscosity and fracture stress.

### 3.6. Compression, Preservation, and Packaging

Compression is the process of stabilizing the tofu gel network structure. The pressing operation applies pressure to the formed curd, expelling the excess yellow water and reducing the syneresis of the tofu during the storage process. During the pressing process, the ratio of the β-sheet structure of soy protein increases, the disordered structure decreases, and the gel system gradually stabilizes [72]. The amount of pressure and the pressing time affect the structure of tofu. The moisture around the soy protein gel network cannot be completely released under low pressure, which leads to uneven shaping of tofu and easy syneresis. Under high pressure, the gel structure of tofu is greatly damaged, resulting in excessive loss of yellow water. Studies have shown that the water retention of tofu is negatively correlated with the loss of yellow water [100], and a certain amount of soluble protein and other nutrients are also dissolved in yellow water. Therefore, once the amount of yellow water decreases during the pressing process, the water retention and nutritional value of tofu also decrease [101]. Tofu is preserved by adding preservatives to increase product shelf life [102]. Tofu preservation requires a comprehensive preservation technology. Freezing can achieve the effect of freshness, and the freezing temperature and time greatly influence the texture of tofu. Freezing treatment converts the water in the tofu into ice crystals, expands the mesh inside the original tofu tissue, and improves the texture characteristics of the tofu. However, a very low temperature makes the ice crystals in the protein gel network too dense, and the pores become smaller after thawing, which can decrease the texture quality of tofu [103]. Kobayashi et al. [104] found that the ice recrystallization and dehydration of frozen tofu with a shelf life of 0–7 days causes changes in the balance of hydrophilic and hydrophobic zones. This change induces the formation of new protein interactions, resulting in a firmer tofu texture.

Tofu packaging is the last step in tofu processing. The choice of packaging materials affects the water retention and shelf life of tofu. Tofu is rich in water and protein, which can deteriorate rapidly. Appropriate packaging can prevent the growth of microorganisms and slow down protein deterioration and water loss, thereby extending the shelf life of tofu [105].

## 4. Conclusions

The production of tofu includes a series of processes, such as soybean screening, soaking, grinding, filtering, boiling, coagulating, pressing, preserving, and packaging. The composition, structure, and content of soy protein are constantly changing during the production process. The gelation properties of soy protein are the basis for the preparation of tofu.

Different soybean varieties have different genotypes, protein composition, 11S/7S protein ratio, etc. The growth environment and storage conditions of soybeans also have a significant impact on the composition and structure of the protein. Soybeans become raw soymilk after soaking and grinding, and soy protein is dissolved from the solid phase to the liquid phase to form an emulsion. In the boiling process, the soymilk protein is denatured and the hydrophobic groups are exposed. By adding a coagulant to soymilk, a curd network structure with protein as the backbone is formed, and the pressing process stabilizes the curd network structure. The subsequent preservation and packaging operations further affect the structure of the curd.

The soymilk system is complex and changeable. Tofu products are mature and diverse. The research in this field covers a wide area, but it is not easy to go deep. The composition structure and spatial configuration of soy protein have not yet been fully resolved. At present, analyses of the interaction between the components and the curdling process are based on model predictions rather than actual observations. Most of the established models require adjustments because there are still contradictions between different models.

We suggest that further clarifying the curd formation mechanism using different coagulants and analyzing the changes at a molecular level can contribute greatly toward improving tofu quality. In addition, the specific changes that occur during the interaction and assembly of soy protein, oil, phytic acid, and other components in the curd are also an important research direction. The application of molecular simulation technology in the analysis of the composition and structural changes of soybean protein and other important components should be the focus of future research.

## Figures and Tables

**Table 1:** Composition, structure, and physiological functions of soy protein [5,6,7,8,9,10]; Protein Data Bank.

Component	Ingredient	Structure	pH	Relative Molecular Mass	
				A	B
2S (15~22%)	Trasylol	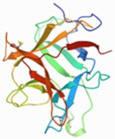	4.5	8000~21,500	15,000~30,000
	Cytochrome C	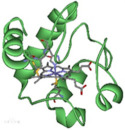	10.2~10.8	12,000	
7S (34~37%)	Hemagglutinin	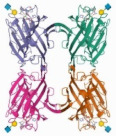	-	102,000	100,000~200,000
	Lipoxygenase	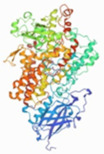	5.7~6.4	102,000	
	β-amylase	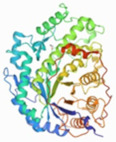	5.0~6.5	61,700	
	β-Conglycinin	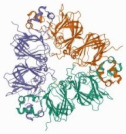	5.07~5.88	180,000~210,000	
11S (31~42%)	Glycinin	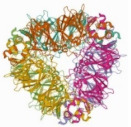	5.28~5.78	350,000	350,000
15S (9~10%)	-	-	-	600,000	600,000

-: Not Available.

**Table 2:** The composition and functions of 7S and 11S [15,16].

Protein	Subunit	Molecular Weight (kDa)	Isoelectric Point
7S β-Conglycinin	α	57~72	5.23
	α’	57~68	5.07
	β	45~52	5.88
11S Glycinin	A1aB1b	53.6	-
	A2B1a	52.4	-
	A1bB2	52.2	-
	A5A4B3	61.2	-
	A3B4	55.4	-
	A1a	-	5.78
	A1b	-	5.28
	B1a	-	5.46
	B1b	-	5.73
	A2	-	5.46
	A3	-	5.60
	A4	-	5.29

-: Not Available.
